# Is the time right for transplanting immature testicular tissue or cells to restore male fertility? Expert perspectives on clinical implementation of autotransplantation of cryopreserved testicular tissue or cells for fertility restoration

**DOI:** 10.1016/j.bpobgyn.2025.102638

**Published:** 2025-07-11

**Authors:** Myriam Safrai, Ellen Goossens, Rod T. Mitchell, Kyle E. Orwig, Callista L. Mulder, Ans M.M. van Pelt, Debra A. Gook, Aurélie Feraille, Emily Delgouffe, Jill P. Ginsberg, Jan-Bernd Stukenborg, Kathleen Duffin, Kirsi Jahnukainen, Claus Yding Andersen, Marianne D. van de Wetering, Michael P. Rimmer, Virginie Barraud-Lange, Nina Neuhaus, Sheila Lane, Hooman Sadri-Ardekani, Nathalie Rives, Christine Wyns

**Affiliations:** a IVF Unit, Department of Obstetrics and Gynecology, Chaim Sheba Medical Center (Tel Hashomer), Sackler Faculty of Medicine, Tel Aviv University, Tel Aviv, Israel; b Biology of the Testis (BITE) Laboratory, Research Group Genetics, Reproduction and Development (GRAD), Vrije Universiteit Brussel, (VUB), 1090, Brussels, Belgium; c Centre for Reproductive Health, Institute of Regeneration and Repair, University of Edinburgh, Edinburgh, UK; d Royal Hospital for Children and Young People, Edinburgh, UK; e Department of Obstetrics, Gynecology and Reproductive Sciences, Magee-Womens Research Institute, University of Pittsburgh School of Medicine, Pittsburgh, PA, USA; f Reproductive Biology Laboratory, Center for Reproductive Medicine, Amsterdam UMC Location University of Amsterdam, Amsterdam, the Netherlands; g Amsterdam Reproduction and Development Research Institute, Amsterdam, the Netherlands; h Reproductive Services, The Royal Women’s Hospital, Parkville, VIC, Australia; i Department of Obstetrics and Gynaecology, Royal Women’s Hospital, University of Melbourne, Parkville, VIC, Australia; j NorDIC, Team “Adrenal and Gonadal Pathophysiology, ” Biology of Reproduction-CECOS Laboratory, Rouen University Hospital, Rouen Normandy University, Rouen, France; k Division of Oncology, Children’s Hospital of Philadelphia, Department of Pediatrics, University of Pennsylvania Perelman School of Medicine, Philadelphia, PA, USA; l NORDFERTIL Research Lab Stockholm, Childhood Cancer Research Unit, Department of Women’s and Children’s Health, Karolinska Institutet, and Karolinska University Hospital, Solna, Sweden; m NORDFERTIL Research Lab Uppsala, Department of Organismal Biology, Uppsala University, Uppsala, Sweden; n Division of Haematology-Oncology and Stem Cell Transplantation, New Children’s Hospital, Pediatric Research Center, Department of Pediatrics, University of Helsinki and Helsinki University Hospital, Helsinki, Finland; o The Fertility Clinic and the Urological Department Herlev University Hospital of Copenhagen & Faculty of Health and Medical Sciences, University of Copenhagen, Copenhagen, Denmark; p Princess Maxima Center for Pediatric Oncology, Utrecht, the Netherlands; q Department of Reproductive Biology CECOS, Public Assistance-Paris Hospital (AP-HP) Paris Centre University Hospital (HUPC), Cochin Hospital, Paris, France; r AYA Unit, Fertility Preservation Consultation, Haematology Department, Public Assistance-Paris Hospital (AP-HP), Paris North University Hospital (HUPN), Nord, University of Paris Cité, Saint-Louis Hospital, Paris, France; s Centre of Reproductive Medicine and Andrology, University of Münster, Münster, Germany; t Nuffield Department of Women’s & Reproductive Health, University of Oxford, England, UK; u Institute of Regenerative Medicine and Department of Urology, Atrium Health Wake Forest Baptist, Winston-Salem, NC, 27157, USA; v Department of Gynecology and Andrology, Cliniques Universitaires Saint-Luc, Université Catholique de Louvain, Brussels, Belgium

**Keywords:** Fertility preservation, Immature testicular tissue, Spermatogonial stem cell transplantation, Pre-pubertal boys, Testicular tissue cryopreservation, Testicular tissue transplantation

## Abstract

Transplantations of cryopreserved immature testicular tissue (ITT) or spermatogonial stem cells (SSCs) represent promising approaches to restore fertility in patients with testicular tissue or cell suspension cryopreserved for fertility preservation. With over 3000 testicular samples cryopreserved globally, clinically viable and standardized protocols restoring fertility using cryopreserved testicular tissue/cells are urgently needed. Decades of research demonstrate the feasibility of immature testicular tissue transplantation (ITTT) or spermatogonial stem cell transplantation (SSCT) in animal models, including non-human primates. However, significant challenges remain in translating these options to human clinical practice.

Critical factors include rigorous patient selection, robust pre-transplant evaluations to mitigate risks of malignant cell reintroduction, and the optimization of transplantation timing to support spermatogenesis. Developing comprehensive follow-up protocols and international data-sharing frameworks is essential to optimize outcomes. While offering the potential for genetic parenthood and enhanced quality of life for cancer survivors, these techniques require further refinement to ensure safety, efficacy, and realistic expectations. This paper outlines the framework for advancing the clinical translation of autotransplantation of testicular tissue/cells through collaboration and innovation.

## 1. Introduction

Thirty years have passed since the group of Ralph Brinster first laid the experimental groundwork for unlocking the potential of mouse spermatogonial stem cells (SSCs) to regenerate spermatogenesis in infertile recipients and produce offspring [1,2]. In addition, over twenty years have passed since the first successful grafting of immature testicular tissue (ITT) fragments, resulting in spermatogenesis and the production of offspring in a mouse model [3–5]. These successful approaches in animal models have strengthened the idea that SSCs may provide a means to treat human male infertility, especially in those at risk of infertility due to a gonadotoxic treatment in childhood.

SSCs are the basis for lifelong spermatogenesis, a process in the testes from puberty onwards. SSCs can self-renew and differentiate if adequately supported by a functional microenvironment [6,7]. SSCs and their microenvironment are sensitive to various exposures, such as chemotherapy and radiotherapy [8,9], making infertility a common long-term side effect in adult survivors of childhood cancer or those undergoing bone marrow transplantation for treatment/cure of non-malignant disease (e.g., sickle cell disease, β-thalassemia). SSCs are present in the testes from birth onwards [10]. This provides an opportunity to preserve fertility before highly gonadotoxic treatment commences by performing a testicular biopsy and cryopreserving testicular tissue containing viable and transplantable SSCs [11]. The first descriptions of successful ITT transplantations (ITTT) leading to live offspring were conducted in mice, and further progress has been made toward human clinical application for both spermatogonial stem cell transplantation (SSCT) and ITTT. Both strategies have been effective in various animal models, including non-human primates (NHP), using autologous [12] and allogeneic transplants of testicular cells [13,14], ITT autografts [15,16], and xenotransplantation [17,18]. Lessons learned from experiments in animals highlight the remaining challenges for clinical application(s) [19]. More importantly, achievements in NHP and xenografting experiments with human ITT suggest that these methods could yield mature sperm in future clinical approaches [15, 17,20,21]. Additionally, studies for both recipients and offspring have been conducted following SSCT in mice [20,22,23], whilst ITTT in NHP has resulted in the birth of a single healthy monkey [15].

Studies aimed at translating autotransplantation into clinical application are limited. A proof-of-concept study to establish a propagation system for human SSCs has been developed to increase the efficiency of the SSCT approach for future clinical trials [24]. Furthermore, a recent study reported the autologous grafting of frozen-thawed adult testicular tissue in a man with non-obstructive azoospermia. Graft survival was observed six months post-transplantation; however, sperm were not identified in the grafts [25].

Cancer survivors who achieve paternity report significantly better long-term quality of life (QoL) across various domains, including social and emotional functioning, general QoL, and treatment satisfaction [26]. This highlights the growing need for clinical implementation of SSCT and ITTT to address hopes of increasing numbers of childhood cancer survivors wishing to build their own families [27]. To enable the possibility of future genetic parenthood, some clinics have offered testicular tissue cryopreservation for more than twenty years [11,28]. It has been reported that over 3000 testicular biopsies are currently in cryo-storage worldwide [11]. This number is likely an underestimation, and the number of patients offered testicular tissue cryopreservation rapidly increases. Therefore, the medical community is anticipating a growing number of adult patients who are permanently infertile following gonadotoxic treatment and requesting the use of their cryopreserved ITT. While testicular tissue cryopreservation protocols are well-described [21,29–31], there remains an urgent need for clinically applicable fertility restoration methods.

Several centers worldwide have already obtained ethical approval to perform SSCT and ITTT [11]. Collaborative efforts are being made to identify the critical factors and best practices for successfully translating these techniques into clinical practice. As an initiative led by the ORCHID-NET consortium (https://www.orchid-net.com/), an international network of experts in the field of male fertility preservation, this expert opinion paper aims to offer our perspectives on the optimal clinical implementation of SSC-based fertility restoration treatments, with a focus on ensuring eligibility, feasibility, safety, and efficacy for patients.

## 2. Pre-transplant patient selection and management

### 2.1. Patient selection

Careful patient selection is crucial for successful fertility restoration through SSCT or ITTT.

At cryopreservation, eligibility focuses on long-term infertility risk and immediate clinical context. At the time of transplant, the focus shifts to the patient’s current infertility status, tissue viability, absence of residual disease, and the patient’s (and guardians, when relevant) wishes. The selection process should consider each patient’s underlying condition, the absence of ongoing spermatogenesis with clear evidence of male infertility, the potential for fertility restoration, and the risks involved in the procedure. Proposed eligibility criteria include: 1) patient cryopreserved testicular tissue for fertility preservation; 2) semen analysis indicates that patient is azoospermic; 3) patient has completed gonadotoxic treatment and is a healthy survivor of their primary disease; 4) consistency with pre-transplant testing (as outlined below) in addition to institution-specific requirements.

### 2.2. Pre-transplant testing requirements for SSCT or ITTT

#### i Assessment of testis function and reproductive potential of the patient

Evaluation of the fertility status of the patient should be confirmed before grafting to avoid unnecessary ITTT or SSCT. This evaluation would involve standard clinical work-up including physical examination, hormone assessment, and semen analysis, which are included in standard investigations for male factor infertility [32,33].

Physical examination includes the determination of testicular volume with an orchidometer. Low testis volume (<15 mL) can be indicative of spermatogenic failure, which should be confirmed by semen analysis.

As sperm production is variable daily, semen analyses should be performed on at least two occasions, with 2–7 days of abstinence and 2–3 months between analyses. In the case of azoospermia in the standard semen analysis, the complete absence of ejaculated sperm should be further confirmed by extensive semen examination after centrifugation and observation of the whole pellet according to WHO criteria [34].

Serum hormone levels, particularly FSH, LH, and testosterone, are important in assessing testicular function and should be measured and can serve both as a diagnostic tool for azoospermia and as a reference baseline for follow-up evaluations after transplantation. Additional hormones such as AMH and inhibin B may be explored to assess whether they provide supplementary information in specific clinical contexts. Hypergonadotropic hypogonadism following gonadotoxic therapy indicates direct damage to the testis; however, there are no absolute thresholds to predict the absence of spermatogenesis or surgical sperm retrieval rates. In cases of cranial irradiation, patients may present with hypogonadotropic hypogonadism, and in these cases, the fertility potential can only be evaluated after hormone stimulation with gonadotropins [35].

Testicular ultrasound examination is an integral part of the infertility diagnostic and pre-operative work-up and to exclude the presence of a tumor. In the context of SSCT, a standard ultrasound examination can determine the optimal site for SSCT. The ideal ultrasound findings include a homogenous testicular image with a visible sonographically dense mediastinum (rete testis), which has been identified as the most suitable site for testicular cell injection in large testes [36,37]. Pre-transplantation testicular ultrasound is also essential for further testicular growth monitoring and detecting potential complications after SSCT or ITTT. In addition to the clinical evaluation, the history of testicular irradiation should be noted as it may impair the somatic environment and vascularization necessary to support graft survival and spermatogenesis, potentially compromising the success of transplantation.

#### ii Assessment of the fertility potential of cryopreserved ITT

The assessment of the testicular biopsy obtained during cryopreservation should focus on the presence of spermatogonia, evaluating the reproductive potential of the stored tissue. Based on single-center cohorts of patients who underwent a testicular biopsy for fertility preservation, the presence of spermatogonia was confirmed in 95–100 % of cases [38,39]. In cases where no spermatogonia are observed at histology and histochemistry with markers such as MAGEA4 or DDX4, spermatogonia might be present in other cryopreserved testicular fragments when testis tissue is not homogeneous. However, in such cases, and when congenital germ cell aplasia is suspected, careful consideration of the eligibility for transplantation is warranted. In this context, thawing additional fragment(s) of tissue fragments is recommended.

#### iii Evaluating the microbiological safety of cryopreserved tissue

Generally, a microbiological analysis of the transport and freezing medium during testicular tissue cryopreservation is performed. If not, the absence of bacterial contamination can be evaluated before SSCT or ITTT [40]. If bacteria are detected, expert advice is recommended to assess the infectious risk and potential therapeutic approach to prevent infection of the recipient.

#### iv Additional requirements specific for childhood cancer survivors

SSCT and ITTT in cancer survivors require a thorough risk assessment to mitigate the potential reintroduction of malignant cells [41,42]. Hematological malignancies, such as leukemia and lymphoma, pose a significant risk due to the possible presence of circulating malignant cells. Overall contamination rates of 37 % were observed in the testes of pre-pubertal patients suffering from leukemia and lymphomas, varying with the stage of the disease, the time of assessment relative to the treatment phase, and the detection technique [41]. In a series of pre-pubertal and peripubertal patients who underwent ITT preservation before the start of any chemotherapy or radiotherapy for hematological cancers, contamination rates were as high as 39 % (57 % for ALL and 14 % for lymphomas), and use of polymerase chain reaction (PCR) to detect specific cancer cell markers (T-cell rearrangements and clonal immunoglobulin) proved to be superior to histology and immunohistochemistry [43,44]. Furthermore, some solid cancers present a risk of testicular involvement, including rhabdomyosarcoma, melanoma, and testicular germ cell tumors. Even malignancies deemed low-risk, such as certain sarcomas or neuroblastoma, may infiltrate the testes, emphasizing the necessity for comprehensive assessment before transplantation [45].

A rigorous risk assessment process utilizing multiple diagnostic modalities is essential to detect the presence of minimal residual disease (MRD) within the cryopreserved testicular tissue. Conventional anatomopathological analysis remains the cornerstone, allowing for the detection of abnormal cell pathology and malignancy-associated features. While immunohistochemical analysis can detect specific tumor markers, offering enhanced specificity in identifying malignant cells, molecular techniques like PCR enable highly sensitive detection of molecular markers, possibly detecting a minimal amount of residual disease. Complementary diagnostic methods, including flow cytometry and *in-vivo* xenotransplantation, can also be used to test a fragment of the cryopreserved testicular tissue or samples of testicular cell suspensions. For xenotransplanted (transplantation from one species into another) material, close monitoring of the recipient animal for several months and subsequent analysis of the graft can provide an additional approach to detect any residual malignant disease [41]. However, it must be emphasized that no matter how detailed the analysis of a sample of the tissue/cells is before transplant, it is impossible to know with absolute certainty that the actual material transplanted is free of malignant cells.

#### v Consent and ethical considerations

SSCT and ITTT present a promising possibility for fertility restoration from cryopreserved ITT. However, these innovative options require consideration of several ethical issues and challenges. Obtaining informed consent, a cornerstone of ethical medical practice, particularly in the context of SSCT and ITTT, is challenging due to the experimental nature of the procedures [46], the impact of their previous medical condition, i.e. malignant risk, cognitive comprehension, and the possible involvement of minors [47] see section 3.1. It is essential to ensure that patients, parents, or guardians fully understand the procedure, including its experimental status, and that outcomes remain uncertain. Clear communication about potential risks, benefits, and current limitations of knowledge is crucial to support an informed decision.

A risk-benefit analysis is a critical component of the decision-making process. For cancer survivors, a major ethical challenge is related to the potential reintroduction of malignant cells in recipients. While the medical team should prioritize the use of advanced screening techniques and carefully weigh the possibility of fertility restoration against the risk of cancer recurrence, it is also paramount to ensure transparency by communicating that no diagnostic technique can guarantee the absence of residual malignant cells. In addition, when testicular tissue cryopreservation is proposed in patients with genetic diseases, assessing the risk of genetic disease transmission is crucial, as genetic anomalies could be transmitted to offspring following SSCT and ITTT. Transparent counseling about the implications and limitations, including the partner’s reproductive potential and alternative reproductive options (e.g., sperm donation or adoption) or preimplantation genetic testing, should be provided to ensure informed decision-making.

## 3. Transplantation protocols

Developing a transplantation protocol is crucial for restoring fertility safely, effectively, and reproducibly. This section highlights key considerations for addressing risks and challenges.

### 3.1. Timing of transplant

Initiation and maintenance of spermatogenesis depend on FSH and LH stimulation, resulting in Sertoli cell maturation and androgen production by Leydig cells, respectively. When considering the timing of transplantation for young patients, there are three possibilities: pre-pubertal, pubertal, and adult. Transplantation in prepuberty has the potential for the transplanted tissue/cells to be exposed to an environment of relative quiescence of the hypothalamus-pituitary-gonadal axis, followed by the gradual increase in pulsatile FSH/LH stimulation that occurs over several years during the pubertal transition phase. Transplantation during puberty or adulthood would immediately expose the transplanted tissue/cells to relatively high levels of FSH/LH. It remains unknown whether testicular grafts are better supported by an immature environment or a mature one, and the optimal timing for transplant may differ for SSCT and ITTT. In addition to this biological uncertainty, psychological maturity and the ability to consent [47] are essential factors in transplant timing and the decision to undergo transplantation should also be guided by the individual’s expressed desire for genetic parenthood. It should be noted that in young patients, deferring transplantation may allow future access to novel fertility technologies that could be precluded once the tissue has been used. Therefore, besides other pre-transplant requirements, we currently recommend that patients should be at least 16 years of age before they are offered a transplant. Importantly, the goal of both SSCT and TTT is fertility restoration. Unlike ovarian tissue transplantation, it remains uncertain whether these procedures can restore meaningful endocrine function, and this aspect requires further evaluation. Therefore, the primary objective remains the reinitiation of spermatogenesis and the potential to achieve biological parenthood. Table 1 highlights key parameters and corresponding challenges to consider regarding the timing of the transplantation procedure.

### 3.2. Infrastructure requirements for tissue transplantation

Infrastructure requirements will depend on country-specific regulations, and compliance with stringent quality control regulations is essential. Cryopreserved testicular tissue is generally stored as small fragments in cryovials using slow freezing with cryoprotectants. [11]. Before transplantation, fragments are thawed in a controlled fashion and prepared in a sterile laminar flow environment. For SSCT, enzymatic digestion is used to produce a cell suspension. For ITTT, thawed tissue fragments are directly transplanted. It is recommended that appropriate sterile techniques for human tissue handling within a laboratory equipped with a laminar flow hood and an incubator for maintaining controlled temperatures are required, preferentially located near the operating theater. This setup facilitates efficient thawing and preparation of tissue for transplantation.

### 3.3. Specificities for the SSCT procedure

#### i Injection site

In the operation theater, an injection pump may be used to control the rate and pressure of the injection. Other options for injection include a syringe or gravity infusion from an IV bag [14]. Access to ultrasound is required to guide the injection needle into the desired location. In mice, three sites have been suggested for SSC injection: efferent ducts, seminiferous tubules, and rete testis [48]. In humans, the efferent ducts are densely coiled and confined within a limited space [49], making them an impractical site for SSC transplantation. Injection into a single seminiferous tubule delivers SSCs only in the injected tubule and filling the entire testis would require injections in multiple tubules. Since many individual tubules with both ends are connected to the rete testis, one single injection into the rete testis can potentially fill the whole testis. However, injection into the rete testis may cause leakage of the transplanted suspension into the interstitial tissue because it is difficult to control the injection angle and depth into the rete testis. In NHP, SSCT was performed into the rete testis using a 25-G spinal needle under slow, constant pressure. The injection was performed under general anesthesia and with ultrasound guidance. The total procedure took 20–30 min for both testes [12,14,37]. Several ex-vivo studies confirmed the efficacy and feasibility of rete testis injections on human cadavers [50,51].

#### ii Quantity and density of cells for transplant

Spermatogenesis was successfully established when adult rhesus macaques were injected with 1000 μL cell suspension. The average cell concentrations of viable testicular cells were 88 × 10^6^ per adult testis and 45.8 × 10^6^ per juvenile testis [14].

In human cadaver testes, injections with 3000 μl of 10 million cells per ml exhibited the best filling rates based on Single Photon Emission Computed Tomography (SPECT) data [50]. Increasing the injection volume is unlikely to enhance the filling and the transplantation efficiency. Higher volumes may result in increased leakage rates, potentially reducing the success of the transplantation. Additionally, injecting a high volume into the testis can cause ischemia and significant damage to the testis.

#### iii SSCT - Transplant as testicular cell suspension or selected SSCs

The quantity of SSCs injected is probably an important factor for the success and efficiency of transplantation [52]. Higher transplantation efficiencies may be obtained if SSC selection has taken place prior to injection; however, this must be balanced against the fact that methods currently available for selecting SSCs may result in cell loss which may reduce transplantation efficiency.

Magnetic-activated cell sorting and fluorescence-activated cell sorting have been employed to enrich SSC populations. Markers that could be employed to select for human SSCs are α6-integrin, GFRα1, THY1 [53,54] TSPAN33, and FGFR3 [55].

An important concern is the risk of reintroducing malignant cells into patients cured of their disease. To address this issue, cell sorting methods have been employed to remove malignant cells prior to transplant. Although, in mouse studies, the results were promising [56], for human SSCs, only limited success was observed, even if using multiple parameters [53,57,58]. Another method to eliminate malignant cells from a cell suspension is culturing the testicular cells in a culture system designed to support the survival of SSCs, whilst eliminating leukemic cells [44]. Given that methods to eliminate malignant cells have not been optimized for human testicular cells/tissue, we recommend cautiously evaluating the risk of re-transplantation on a case-by-case basis in patients with malignancies. In case of expected and known malignant infiltration in the cryopreserved material, transplantation of cells should only be considered if robust methods to eliminate cancer cells become available.

#### iv In-vitro propagation of SSCs

Biopsies retrieved from young boys are limited in size and contain a low number of SSCs. Therefore, the propagation of these cells in-vitro might be necessary. Sadri-Ardekani et al. were the first to report the propagation of human pre-pubertal SSCs [59]. Some studies using similar culture systems were unable to reproduce similar findings [60,61]. One study succeeded in long-term culture of human spermatogonial stem cell-like cells from infant boys under xeno-free conditions [62]. This lack of independent replication highlights the necessity of developing a standardized protocol for propagating SSCs for clinical applications.

Without the benefit of SSC expansion in culture, transplanted SSCs are likely to produce small foci of spermatogenesis in the testes. This may produce insufficient numbers of sperm to appear in the ejaculate. Therefore, it may still be necessary to retrieve rare sperm by microsurgical testicular sperm extraction (micro-TESE) that can be used for intracytoplasmic sperm injection (ICSI), similar to the situation with ITTT.

Although, in mice, no major impact was found on the development and general health of two generations of offspring derived after transplantation of in-vitro propagated SSCs ([20,63] and no major differences in DNA methylation were observed between these and control pups, the use of culture systems in the clinic still requires the demonstration of genetic and epigenetic stability of in-vitro propagated human SSCs.

### 3.4. Specificities for the ITTT procedure

#### i Transplantation site for testicular tissue fragments

In mouse models, the first grafting experiments were performed using ectopic sites. Complete spermatogenesis was reported in dorsal subcutaneous xenografts from several species [4,5,18,64–71] including NHP [15], but not humans [72].

Xenotransplantation of human ITT under the scrotal skin permitted meiotic progression [73], although this did not result in the completion of spermatogenesis. While dorsal subcutaneous grafts of pre-pubertal marmoset tissue showed spermatocytes to be the most advanced germ cells, differentiation into spermatozoa was observed in 33 % of intratesticular grafts [17]. Xenotransplantation of pre-pubertal human testicular tissue into the mouse testis also resulted in better graft survival and germ cell differentiation than subcutaneous grafting. However, full spermatogenesis has not yet been reported [73].

Autologous transplantation of macaque testicular fragments, whether under the dorsal or scrotal skin, showed very high-efficiency development of spermatogenesis with sperm competent to fertilize macaque oocytes by ICSI, leading to the birth of a healthy baby macaque [15]. The graft location did not affect the proportion of tubules showing complete spermatogenesis. Therefore, in a clinical setting, grafting may be performed into the testis or subcutaneously, preferably under the scrotal skin, based on the lower scrotal temperature required for optimal spermatogenesis [74].

#### ii Fragment size

Xenotransplantation experiments on mice were performed with tissue pieces of 0.5–1.5 mm^3^ for grafting into the testis [73,75], 1–6 mm^3^ for grafting under the scrotal skin [76], and 0.5–10 mm^3^ for grafting under the dorsal skin [18,70,72]. However, in macaque, larger tissue fragments of 9–20 mm^3^ could also be successfully transplanted [15]. As testicular tissue is usually cryopreserved in fragments of <10 mm^3^ [11], further tissue fragmentation may not be needed. A better understanding of the tissue dimensions in relation to ischemic damage and site-specific delay to neovascularization is required.

#### iii Number of fragments

No study assessed the optimal number of tissue fragments to be grafted. While multiple human testicular fragments could be transplanted in the scrotum and testis for clinical application, transplanting only part of available tissue fragments to allow for a second transplantation procedure or alternative restoration method later, if required, could be considered. The quality of the cryopreserved tissue (number of SSCs and health of the niche) will influence potential success with a particular volume of tissue, i.e., size and number of fragments.

#### iv Surgical approaches for fertility assessment and graft placement

Different surgical approaches could be considered. During a one-step procedure, the graft is performed at the time of fertility assessment by TESE. During a two-step procedure, the graft is performed at a later time if no spermatozoa is retrieved at TESE. Consideration on both approaches are presented in Table 2.

## 4. Follow-up for patients

Post-transplantation follow-up is crucial to ensure patient safety and optimize outcomes for this innovative procedure. Over the years, a tissue transplant has been reported following adult tissue harvesting [25]. However, no standardized protocols for post-transplantation follow-up currently exist. Therefore, we propose a comprehensive follow-up protocol to systematically gather data among researchers and clinicians using standardised outcome measures. This includes the creation of a robust database to generate evidence for refining practices and optimizing outcomes, and eventually improve the quality of care for these patients.

We recommend initiating follow-up monitoring at regular intervals (e.g. 1, 3, and 6 months) post-transplantation, followed by annual evaluations. Long-term follow-up, extending to 10 years or more, is essential to monitor sustained functionality and detect late-onset complications and potential recurrence of malignancy. Each follow-up should include the following:

- Hormonal assessments, including FSH, LH, testosterone, inhibin B, and AMH serum levels.
- Ultrasound to assess testicular volume, structural changes such as fibrosis, and graft volume.
- Following SSCT, the follow-up should include a semen analysis. Following ITTT, data on sperm retrieval rate from the graft should be reported according to the transplantation site.
- Reproductive outcomes, including fertilizing capacity and embryo development plus information of pregnancies and their outcomes (e.g. miscarriage and live births), and appropriate follow-up of the health of the children.
- In cases where the procedure is performed in cancer survivors, any signs of disease relapse should be closely monitored and evaluated. The patient should be informed that in the case of disease relapse, removal of the transplanted tissue is necessary.
- In case of the absence of sperm in the ejaculate following SSCT, any data on additional procedures such as TESE or microTESE and their outcomes should be collected.
- Following SSCT or ITTT, QoL of the patient should be monitored based on available questionnaires.
- Follow-up data on transplantation should ideally be collected in the form of an international patient registry.

## 5. Timing and strategy for ITT graft retrieval

The goal of ITTT is the retrieval of functional sperm for use in assisted reproduction. Unlike SSCT, sperm are not expected to appear in the ejaculate following ITTT, and thus graft assessment relies on direct tissue evaluation. At present, there are no standardized guidelines on the optimal timing for graft retrieval. Data from non-human primate studies suggest that complete spermatogenesis within grafts may take 6–12 months to establish, though inter-individual variation is substantial [15]. In human applications, the time to detectable sperm production remains undefined. Serial hormonal profiling may offer indirect signs of graft activity, but definitive evidence requires histological confirmation.

We propose surgical retrieval of grafts at 6–18 months post-transplantation, guided by clinical and hormonal parameters. If more than one graft emplacements have been done, we suggest a partial removal of the transplant to allow further maturation if no sperm is retrieved. Histological analysis can confirm the presence of mature sperm, and, when indicated, microdissection testicular sperm extraction (microTESE) should be performed to recover sperm for intracytoplasmic sperm injection (ICSI). In the absence of sperm, cryopreservation of graft-derived tissue may still preserve the potential for future use. In the future, establishing clear criteria and timelines for graft retrieval will be essential for optimizing outcomes and minimizing unnecessary procedures in clinical TTT implementation.

## 6. Summary

The impact of ITTT and SSCT on patient QoL is potentially transformative. For childhood cancer survivors, the ability to achieve genetic parenthood has the potential to enhance long-term emotional and psychological well-being. While decades of experimental research have yielded promising preclinical results for SSCT and ITTT, the absence of standardized protocols for clinical application remains a significant hurdle. Developing these protocols is crucial to ensuring safety, consistency, and efficacy in clinical practice.

A major challenge lies in the limited availability of ITT for research and the limited number of patients per center, which restrict progress in refining these techniques. Enhanced collaboration between researchers and clinicians based on well-defined protocols is essential to address these limitations, driving innovation and facilitating the translation of preclinical findings into viable clinical applications.

After presenting knowledge on outcomes of preclinical studies, this expert opinion paper emphasizes stringent patient selection, thorough pre-transplant assessments and alternative protocols for the procedures, and recommends robust follow-up to ensure safety and optimize outcomes.

In conclusion, with coordinated global efforts, this field holds immense potential to revolutionize fertility preservation and improve the lives of many patients worldwide.

Practice Points:

- Testicular tissue cryopreservation can be offered to pre- and peri-pubertal patients facing gonadotoxic treatments as part of an approved protocol.
- Re-transplantation of testicular tissue or SSCs may provide an option for restoration of fertility, but this remains unproven.
- Re-transplantation should only be conducted as part of an ethically approved research study.
- Careful assessment of tissue and consideration of the risk of reintroducing malignancy are key considerations.
- Adverse event and repoductive outcomes (Sperm production, embryo development, pregnancy and livebirth) following transplant should be accurately recorded to ensure efiicacy of the procedure.

Research Agenda:

Future research priorities include the following:

- Developing reliable in-vitro SSC propagation methods, refining transplantation techniques, and investigating long-term genetic and epigenetic stability when using sperm generated after SSCT and ITTT.
- Developing methods to remove malignant cell contamination in ITT.
- Identifying non-invasive clinical or biological markers that can reliably predict ongoing spermatogenesis before and following tissue and cell transplantation.
- Identifying the typical timeframe for restoring spermatogenesis following transplantation and determining the optimal timing for graft recovery following ITTT.
- Transplantation of non-propagated cell suspensions from frozen biopsies (without malignancy) directly into the testis to avoid any issues with changes imposed by the *in vitro* culture. Identifying spermatogonial subtypes with the highest potential for implantation, colonization and differentiation.

## Figures and Tables

**Table 1:** Comparative evaluation of key parameters across developmental stages for spermatogonial stem cell and testicular tissue transplantation.

Parameter	Pre-pubertal	Adolescent	Adult
**Parenthood readiness and decisional process**	Parenthood readiness absent. The child needs to make a decision and project himself at a distant point in the future. Decisional autonomy is limited due to the involvement of a guardian.		Immediate parenthood goal may be known and autonomous decisional process possible. **Note:** The age and ovarian reserve of a female partner may be important to consider when deciding the timing of transplantation.
**Hormonal environment**	Normal pre-pubertal hormonal levels (decreased FSH +LH)	Normal pubertal hormonal levels, with potential for elevated FSH or hypogonadotropic hypogonadism	Most often hypergonadotropic hypogonadic pattern (increased FSH±LH) or hypogonadotropic hypogonadism (decreased FSH + LH)
**Treatment completion & remission**	May not allow enough time for treatment completion and adequate remission		Allows for sufficient time for treatment completion and adequate remission
**Fertility assessment (pre-transplantation)**	Cannot be assessed due to immaturity of the gonads	Limited ability to evaluate fertility due to ongoing development*	Fertility status can be reliably assessed*
**SSCT** **Time for spermatogenesis following transplantation**	It is likely that the time for SSC to generate sperm will be extended due to the requirement for gonadotropins to mature the stem cell niche.	The SSC niche being most likely already mature, it is possible that a shorter time will be necessary for the transplant to generate sperm.	
**Relevant to all ages**	1 Uncertainty about a potentially damaged stem cell niche following gonadotoxic therapy. 2 Definitive confirmation that sperm production originates exclusively from the transplanted cells remains unattainable due to the potential for late recovery of endogenous spermatogenesis after gonadotoxic therapies.		
**ITTT** **Time for spermatogenesis following transplantation**	Graft function duration may be limited and may not support the completion of spermatogenesis.		Ready to conceive when sperm is generated.
**Relevant to all ages**	1 Uncertainty about graft survival, the time required for sperm production, and the timing of sperm recovery within the graft. 2 Graft recovery and sperm cryopreservation may be required before parenthood desire.		

FSH: Follicle-Stimulating Hormone; LH: Luteinizing Hormone; AMH: Anti-Müllerian Hormone; SSCT: Spermatogonial Stem Cell Transplantation; ITTT: Immature testicular Tissue Transplantation.

* Hypogonadotropic hypogonadism may be present following brain irradiation, requiring gonadotropin treatment to assess fertility.

**Table 2:** Comparison of one-Step and two-Step approaches for testicular tissue transplantation.

	Pros	Cons
One-step	- Single surgical procedure and related risks - Possibility to directly use or cryopreserve sperm (if found at TESE) while following graft development to optimize the procedure	- Impossible to fully assess the patient’s fertility potential before the decision to transplant - Decision process may be more difficult for the patient, and must be made in advance - Advanced thawing of tissue regardless of testicular sperm found to reduce anesthetic time
Two-steps	- May avoid an additional invasive procedure if testicular sperm is found - Possibility of preparing the grafting site for the second surgery if no sperm is found - Alignment in timing of tissue thawing	- Two invasive surgical procedures with related risks - Presence of scar tissue from previous TESE
